# Symptomatic Cushing syndrome and hyperandrogenemia revealing steroid cell ovarian neoplasm with late intra-abdominal metastasis

**DOI:** 10.1186/1472-6823-14-12

**Published:** 2014-02-08

**Authors:** Menghua Yuan, Mingcai Qiu, Mei Zhu

**Affiliations:** 1 Postal address: Department of Endocrinology, General Hospital of Tianjin Medical University, NO. 154 Anshan Road, Heiping District, Tianjin 300052, China

**Keywords:** Steroid cell ovarian neoplasm, Not otherwise specified, Intra-abdominal metastasis, Cushing syndrome, Hyperandrogenemia

## Abstract

**Background:**

Steroid cell tumors of ovary account for less than 0.1% of all ovarian tumors and these tumours may present at any age in association with interesting presentations related to hormonal activities. The subtype, not otherwise specified (NOS), is associated with androgenic changes in 56-77% and Cushing syndrome in 6-10%. Due to the rarity of available data regarding these tumors, little is known about their malignant potential and metastatic behaviour. We hereby report an unusual metastasis of steroid cell ovarian neoplasm presented with both Cushing syndrome and hyperandrogenemia.

**Case presentation:**

A 31-year-old woman, who had a past medical history of ovarian tumor resection (left ovarian thecoma was initially diagnosed at that time), presented with hirsutism, hypertension and menstrual disorder. Also, laboratory work-up revealed hypercortisolism and androgen excess. Computerized tomography (CT) of the abdomen showed abdominal paraaortic masses, multiple intrahepatic nodules and retroperitoneal lymph nodes enlargement. Positron emission tomography/computed tomography (PET/CT) scan demonstrated metastatic lesions. Her ovarian tumor sections were re-examined and pathology result was corrected to steroid cell tumor (NOS) associated with active cell growth and necrosis. Subsequent excision of metastatic lesions yielded clinical improvement promptly and metastasis of steroid cell tumor was confirmed by postoperative pathological studies. However, one year after the surgical management of metastasis, recurrence happened while radiotherapy was ineffective. The patient finally died of tumor metastatic recurrence.

**Conclusion:**

This case reports a rare coexistence of Cushing syndrome and hyperandrogenemia which occurs based on metastasis of steroid cell ovarian neoplasm. It presents a real diagnostic challenge to both clinicians and pathologists. Therefore, it is very important to establish a final diagnosis by pathological studies along with clinical manifestations and imaging findings. Besides, it is necessary to improve follow-up of patients with this kind of tumors.

## Background

Steroid cell tumors (SCTs) of the ovary are a rare subgroup of sex cord-stromal tumors (SCSTs), representing less than 0.1% of all ovarian tumors [1] and usually occurring in adults with an average age at diagnosis of 47 years old [2]. These tumors can produce steroids and may give interesting presentations related to hormonal activities [3-10]. There are three subtypes of such tumors based on cell of origin: stromal luteoma arising from ovarian stroma, Leydig cell tumor arising from Leydig cells in the hilus, and steroid cell tumor (not otherwise specified, or NOS) when the lineage of the tumor is unknown [1]. The last subtype is usually associated with androgenic changes in 56-77%, estrogen secretion in 6-23%, and Cushing syndrome in 6-10%.

Due to the rarity of available data regarding SCTs, little is known regarding their malignant potential and metastatic behaviour. So far, very few cases have been reported on a late metastatic lesion producing steroid hormones without evidence of recurrence of the primary tumor. Here, we present a rare case of intra-abdominal metastasis of ovarian steroid cell tumor (NOS) which secreted both cortisol and androgen and caused Cushing syndrome and hyperandrogenemia 3 years after the initial tumor was removed.

## Case presentation

A 31 year-old-woman who had a past history of left oophorectomy was admitted to our hospital with marked hirsutism and menstrual disorder as well as significant hypertension.

Three years and six months before the admission, the patient had irregular menses and excess hair growth on her face, neck, chest, abdomen and thighs. Her face became round. Her skin became thin and bruised easily. Then she turned to a local clinic where physical examination showed that she had a blood pressure of 160/120 mmHg. Meanwhile, gynecologic ultrasonography demonstrated a 25 × 20 × 15 cm left ovarian mass. Routine laboratory workup revealed the following: leukocytosis (11.2 × 10^9^/L) with neutrophilia (76.6%), hypokalemia (3.3 mmol/L) and elevated fasting glucose (6.4 mmol/L). Hormonal assays were not conducted. She received left oophorectomy and pathology result was luteinized thecoma of the ovary with focal coagulative necrosis and calcification. Since the patient desired future fertility strongly, she refused total abdominal hysterectomy with contralateral oophorectomy. Postoperatively, the patient’s symptoms of hirsutism and round face were resolved. Her menstrual period and blood pressure returned to normal. Two year later, she gave a natural childbirth and had no difficulty in breast-feeding.

Four months prior to admission, the patient had hirsutism and menstrual disorder again. Simultaneously, she noted a left abdominal mass. From then on, she found her face became round gradually. Her blood pressure went up to 180/110 mmHg, resulting in blurred vision. Her body weight dropped by 3 Kg druing four months while her height did not change. A mass (11 × 8.1 × 5.6 cm) beside abdominal aorta was detected by ultrasound. Lab tests showed her tumor markers (serum Ca-125, Ca19-9, Ca153, CEA and AFP) and 24-hrs urinary VMA were within normal limits. Antihypertensive drug Doxazosin 4 mg/d was given, but her hypertension was not well controlled. For further diagnosis and treatment, the patient was admitted to our hospital.

During physical examination her blood pressure was 195/130 mmHg. She was 147 cm tall and weighed 54 kg (body mass index 25.0). She had hirsutism and her skin was thin with scattered ecchymosis. She had a round and plethoric face, central adiposity, buffalo hump and supraclavicular fat pads. A mass of 7 × 5 cm could be palpated in the left mid-abdomen. There were no purple striae and hyperpigmentation.

**Figure 1:**
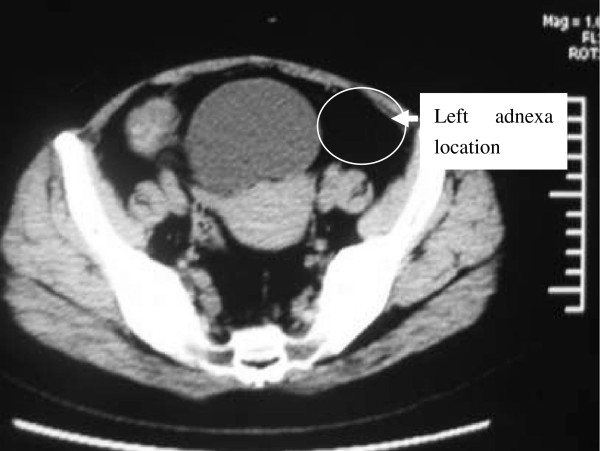
Pelvic CT scan showing no recurrent or residual mass in the left adnexa location, and the right ovary and the uterus were of normal appearance.

**Figure 2:**
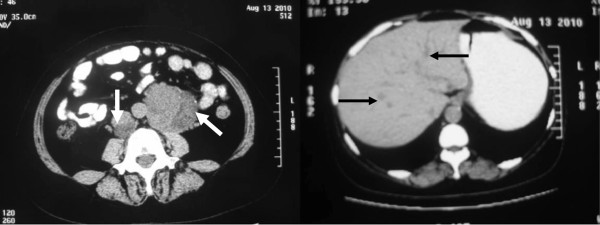
Abdominal CT scan showing two masses (white arrows) beside abdominal aorta, multiple intrahepatic hypodense nodules (black arrows) and retroperitoneal lymph nodes enlargement.

**Figure 3:**
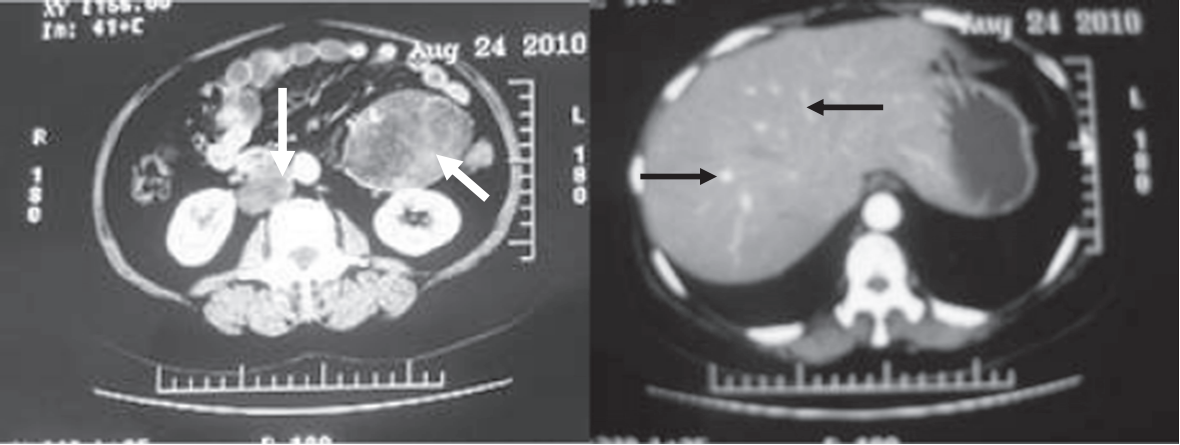
Enhanced abdominal CT scan showing two masses (white arrows), multiple intrahepatic nodules (black arrows) and retroperitoneal enlarged lymph nodes, all enhanced with contrast media.

**Figure 4:**
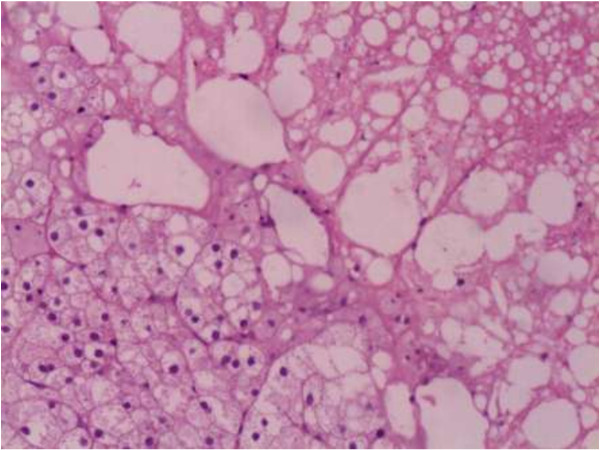
**Histologic section obtained from the ovarian tumor.** Stain: hematoxylin and eosin.

**Figure 5:**
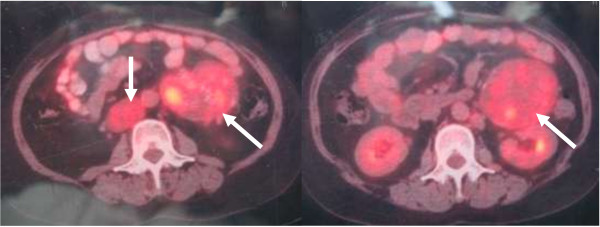
PET/CT revealed abnormally hypermetabolism in the retroperitoneal masses (white arrows) and nodes suggesting metastatic lesions.

Her baseline labs showed normal WBC count (6.66 × 10^9^/L) with neutrophilia (74.7%), hypokalemia (3.11 mmol/L), impaired glucose tolerance (fasting glucose 6.7 mmol/L, 2-hrs postprandial glucose 10.6 mmol/L) and alkalosis (pH 7.54, base excess 9.0 mmol/L). Urine potassium level was within the normal range (50 mmol/24 hr, reference range 25–100) but was relatively high given her hypokalemia. An endocrinological evaluation revealed elevated morning plasma cortisol (31 μg/dl, ref. range 5–25), and overnight dexamethasone (1 mg) failed to suppress plasma level of cortisol. Urinary free cortisol was extremely high (1024.8 μg/24 hr, ref. range 30–110) and plasma ACTH was relatively low (16 pg/ml, ref. range 0–46). In addition, her testosterone level rose to 98 ng/dL (ref. range 14–76) with normal LH, FSH, prolactin and estradiol. Pelvic CT scan showed no recurrent or residual mass in the left adnexa location and the right ovary and the uterus were of normal appearance (Figure 1**)**. However, abdominal CT scan demonstrated two masses beside abdominal aorta, multiple intra-hepatic hypodense nodules, and retroperitoneal lymph nodes enlargement (Figure 2), all enhanced with contrast media (Figure 3). These were compatible with metastatic disease. At the same time, mild adrenal atrophy without ascites was detected in her CT scan. Her previous ovarian tumor sections were re-examined and the pathology result was corrected to steroid cell tumor, NOS (cpinephroma) associated with active cell growth and necrosis (Figure 4). The patient was strongly interested in tumor-reductive surgery. During a pre-surgical work-up, the patient had positron emission tomography/computed tomography (PET/CT) which revealed abnormally hypermetabolism in the retroperitoneal masses and nodes suggesting metastatic lesions (Figure 5). Therefore, she underwent excisions of abdominal paraaortic neoplasms and multiple intra-hepatic nodules as well as retroperitoneal enlarged lymph nodes. The cut surface of the neoplasms showed a solid mass including hemorrhagic and necrotic contents with calcific deposits. The resected specimens showed very similar appearances of histopathology to the previous ovarian tumor. Neoplastic cells were calretinin, CD99, α-inhibin positive, cytokeratin (CK), vimentin weak positive and epithelial membrane antigen (EMA) negative by immunohistochemical staining. These findings were consistent with the diagnosis of malignant steroid cell tumor (NOS) with late intra-abdominal metastasis.

By the 4th postoperative day, her morning plasma cortisol fell to 6 μg/dl while plasma ACTH was up to 41.7 pg/ml. Meanwhile, her plasma testosterone fell to 50 ng/dl and urinary free cortisol fell to less than 36 μg/24 hr (Table 1). During postoperative follow-up (four months after surgery), she showed regression of Cushing syndrome and regained normal menses and blood pressure. Furthermore, her hirsutism disappeared completely. Nevertheless, one year after surgery, metastatic tumors appeared again in the abdominal cavity. Although the patient was given radiotherapy at once, she died of tumor metastatic recurrence sixteen months after surgery.

**Table 1:** Hormone levels before and after surgery

**Hormone**	**Before surgery**	**4 days after surgery**	**Normal values**
Plasma cortisol	31 μg/dl	6 μg/dl	5–25 μg/dl
Plasma ACTH	16 pg/ml	41.7 pg/ml	0–46 pg/ml
Plasma testosterone	98 ng/dl	50 ng/dl	14–76 ng/dl
Urinary free cortisol	1024.8 μg/24 hr	<36 μg/24 hr	30–110 μg/24 hr

## Discussion

We herein present a case of a woman who initially had hirsutism, menstrual disorder and hypertension due to ovarian tumor and developed cortisol and androgen co-secreting intra-abdominal metastasis without evidence of recurrence of the primary ovarian lesion three years after the initial tumor was removed. The tumor was originally diagnosed as luteinized thecoma of the ovary, but at retrospective review the tumor turned out to have subtle features of steroid cell tumor (NOS) such as hypercortisolism and androgen excess.

The most common cause of Cushing syndrome is pituitary or adrenal adenoma. However, in the case of intra-abdominal masses, different etiologies are possible. On the one hand, the patient had some typical clinical features of Cushing syndrome except that she was overweight but thin, which might be caused by underlying devastating malignancy. Her lab tests revealed elevated plasma cortisol which overnight dexamethasone (1 mg) failed to suppress. Thus, Cushing syndrome could be diagnosed. On the other hand, her plasma ACTH was relatively low and the CT images indicated mild adrenal atrophy and intra-abdominal metastatic tumors. As a result, pituitary or adrenal adenoma as well as ectopic ACTH secretion could be ruled out. Based on the above facts and her history of ovarian tumor, paraneoplastic ectopic cortisol secretion was deemed as most likely despite of its rarity. But it was puzzling that ovarian thecoma could not secrete cortisol. Fortunately, her previous ovarian tumor sections were found and the pathological diagnosis of luteinized thecoma was overthrown and corrected to steroid cell tumor (NOS). Ultimately, the patient was diagnosed as malignant steroid cell tumor (NOS) with late intra-abdominal metastasis on the basis of postoperative pathology result. The diagnosis was proved correct by the fact that her symptoms of Cushing syndrome disappeared and her plasma and urinary levels of cortisol decreased significantly after the metastatic lesions were removed. Also in this case no hormone evaluation was performed at the first surgery, but clinical signs strongly suggested co-secretion of cortisol and androgen. Discrepancy between clinics and biology first, and the first result of pathology led to a second pathology reading. Attention should be paid to such a discrepancy by clinicians, in a way that a second reading should have been performed immediately after the first surgery, which should have led to an appropriate and strict follow-up.

In addition, the patient also presented with hirsutism, which might result from hyperandrogenemia rather than hypercortisolemia. Routine screening for Cushing syndrome in women presented with hirsutism is a controversial issue. In a recent study, Karaca et al. screened 105 patients with the main complaint of hirsutism for Cushing syndrome irrespective of their suggestive findings of hypercortisolemia. None of the patients was diagnosed as Cushing syndrome by low-dose dexamethasone suppression test. Therefore, they concluded that routine screening for Cushing syndrome in hirsute patients is not required if the patient does not have accompanying clinical stigmata of hypercortisolism [11].

Sex cord-stromal tumors are developed from the sex cord and stromal components of the gonad. Ovarian SCTs are grouped under SCSTs, and they are usually benign, unilateral, and formed by steroid cell proliferation. Between the three subtypes of SCTs, nearly 60% are steroid cell, NOS tumors. Microscopically, steroid cell tumors (NOS) are generally composed of large, round to polyhedral cells with vacuolated cytoplasm as well as smaller cells with eosinophilic granular cytoplasm, which are usually diffusely arranged in nests, clusters, cords or columns resembling adrenal zona glomerulosa and zona fasciculate. In this case, steroid cell tumor (NOS) was misinterpreted as luteinized thecoma, a typical thecoma throughout which clusters of large eosinophilic, lipid-laden lutein cells are scattered. Luteinized thecoma contains plump spindle cells and therefore is distinguishable from steroid cell tumor (NOS). In addition to these microscopic findings, steroid cell tumors would require immunohistochemical markers for accurate diagnosis. Inhibin and calretinin are the most useful markers for the discrimination of sex cord stromal tumors from other tumors [12]. Most of the steroid cell tumors are positive for calretinin and inhibin [13]. In an immunohistochemical study on 215 ovarian tumors, caleritinin was found to be a sensitive marker for sex cord tumors; however, it was a less specific marker than inhibin in differentiating these stromal tumors from fibrous neoplasms [14]. And sex cord stromal tumors are mostly negative to EMA [12,15]. Histopathologic evaluation for the patient showed inhibin- and calretinin-positive and EMA-negative immunohistochemical findings which made the diagnosis easier. However, some authors have also reported calretinin-negative steroid cell tumors [16,17].

Although most steroid cell tumors (NOS) behave in a benign fashion, malignancy has been reported in as high as 43% of cases [18]. Absolute indication of malignancy is extraovarian metastasis. Furthermore, malignancy is generally associated with identification of the histopathologic findings: two or more mitotic figures per 10 high-power fields, grade 2/3 nuclear atypia, vascular invasion, and a diameter of greater than 7 cm with necrosis or hemorrhage on the gross specimen [1]. The size of the ovarian tumor, presence of necrosis, and nuclear pleomorphism in the present case were suggestive of a malignant nature. Three years later, extraovarian metastasis was confirmed malignancy.

Surgical intervention is the most important and hallmark treatment, and complete excision of the tumor could contribute to the regression of symptoms and disappearance of Cushing syndrome. However, young woman are usually conservative towards this solution due to concern of losing fertility. If future fertility is not an issue, hysterectomy, removal of the contralateral ovary, and complete surgical staging are recommended. Age, large tumor size, lymph node involvement, and residual disease are all predictors of poor prognosis [19]. For stage 2–4 disease, adjuvant radiotherapy or chemotherapy can be implemented, but there are no reports of effective radiation or chemotherapy. An optimal adjuvant chemotherapeutic regiment has not yet been developed, with treatments by the BEP regimen and the carboplatin and paclitaxel regimen yielding equivocal results [20]. Further observation into the nature, biology and behaviour of the tumor may improve the understanding of therapeutic value of radiation or chemotherapy over time.

## Conclusion

This case reports a rare coexistence of Cushing syndrome and hyperandrogenemia which can occur based on ovarian pathology in females. Metastasis of steroid cell ovarian neoplasm resulting in co-secretion of cortisol and androgen is extremely rare and represents a real diagnostic challenge for both clinicians and pathologists. A correct diagnosis usually involves a multidisciplinary effort and neglect of clinical features only results in misdiagnosis. Therefore, it is very important to establish a final diagnosis by pathological studies along with clinical manifestations and imaging findings. Besides, this patient developed steroid-secreting metastasis three years after the excision of primary tumor, and then one year after surgical management of metastasis, recurrence happened while radiotherapy was ineffective. These features of this case illustrate the necessity for this kind of tumor follow-up.

### Consent

Written informed consent was obtained from the patient’s husband for publication of this case report and any accompanying images. A copy of the written consent is available for review by the Editor of this journal.

## Abbreviations

NOS: Not otherwise specified; SCT: Steroid cell tumor; SCST: Sex cord-stromal tumor; ACTH: Adrenocorticotrophic hormone.

## Competing interests

The authors declare that they have no competing interests.

## Authors’ contributions

MY led the acquisition of data, review of literature, and drafted the manuscript. MQ gave the concept and design of research paper. MZ reviewed the manuscript critically. All authors read and approved the final manuscript.

## Pre-publication history

The pre-publication history for this paper can be accessed here:

http://www.biomedcentral.com/1472-6823/14/12/prepub
